# Social value orientation modulates fairness processing during social decision-making: evidence from behavior and brain potentials

**DOI:** 10.1093/scan/nsab032

**Published:** 2021-03-26

**Authors:** Xinmu Hu, Xiaoqin Mai

**Affiliations:** Department of Psychology, Renmin University of China, Beijing 100872, China; Department of Psychology, Renmin University of China, Beijing 100872, China

**Keywords:** social value orientation (SVO), fairness decision-making, P2, medial frontal negativity (MFN), P3

## Abstract

Social value orientation (SVO) characterizes stable individual differences by an inherent sense of fairness in outcome allocations. Using the event-related potential (ERP), this study investigated differences in fairness decision-making behavior and neural bases between individuals with prosocial and proself orientations using the Ultimatum Game (UG). Behavioral results indicated that prosocials were more prone to rejecting unfair offers with stronger negative emotional reactions compared with proselfs. ERP results revealed that prosocials showed a larger P2 when receiving fair offers than unfair ones in a very early processing stage, whereas such effect was absent in proselfs. In later processing stages, although both groups were sensitive to fairness as reflected by an enhanced medial frontal negativity (MFN) for unfair offers and a larger P3 for fair offers, prosocials exhibited a stronger fairness effect on these ERP components relative to proselfs. Furthermore, the fairness effect on the MFN mediated the SVO effect on rejecting unfair offers. Findings regarding emotional experiences, behavioral patterns and ERPs provide compelling evidence that SVO modulates fairness processing in social decision-making, whereas differences in neural responses to unfair *vs* fair offers as evidenced by the MFN appear to play important roles in the SVO effect on behavioral responses to unfairness.

## Introduction

In highly complex social environments, people must consider the outcomes of their own behaviors along with others’ during social interactions; such considerations influence the decision-making process (Rilling and Sanfey, 2011). As per the adage ‘inequality rather than want is the cause of trouble’, fairness norm plays crucial roles in social decision-making. The Ultimatum Game (UG) has been widely employed to examine individuals’ responses to fairness in resource allocation (Güth *et al.*, 1982). Studies using the UG paradigm have revealed that responders are prone to rejecting unfair offers, especially those below 20% of the total (Camerer, 2003; Sanfey *et al.*, 2003; Güth and Kocher, 2014). Rejection of unfair offers contradicts classical game theory, in which the responder should presumably accept any offer because ‘something is better than nothing’ (Morgenstern and Von Neumann, 1953; Rubinstein, 1982). These studies have suggested that people are concerned with their own interests and prefer fairness. When treated unfairly, many people are willing to sacrifice their own interests to punish unfair behaviors in others (Fehr *et al.*, 2002; Decety and Yoder, 2017).

### Theoretical explanations of fairness preferences

Why do humans attach great importance to fairness vis-à-vis resource allocation? Several theoretical models have been proposed to explain individuals’ fairness preferences. Inequity aversion theory holds that people prefer equitable outcomes and are willing to forgo some material payoff in favor of more equitable outcomes (Fehr and Schmidt, 1999; Fehr *et al.*, 2002). Studies using functional magnetic resonance imaging (fMRI) have demonstrated that brain regions associated with rewards, such as the ventral striatum and ventromedial prefrontal cortex (vmPFC), are more active under fair UG offers than unfair offers (Kable and Glimcher, 2007; Tabibnia *et al.*, 2008; Tricomi *et al.*, 2010; Yu *et al.*, 2014).

From an evolutionary perspective, the strong reciprocity model claims that negative reciprocity (i.e. rejecting unfair offers) reflects prosociality because individuals who reject an offer sacrifice their own resources to punish unfair behavior, which may enforce a fair social norm and promote cooperation. This pattern has important evolutionary significance (Bowles and Gintis, 2004; Kaltwasser *et al.*, 2016). Non-invasive brain stimulation studies have reported that disruption of the right dorsolateral prefrontal cortex (rDLPFC) using repetitive transcranial magnetic stimulation or cathodal transcranial direct current stimulation is associated with decreased rejection rates of unfair offers in the UG, indicating that the rDLPFC is involved in exerting cognitive effort to override individuals’ prepotent selfish impulses and thus drives people to consider fairness norms more carefully (Miller and Cohen, 2001; van’t Wout, Kahn *et al.*, 2005; Knoch *et al.*, 2006, 2008; Feng *et al.*, 2015). Moreover, the anterior cingulate cortex (ACC) has demonstrated stronger activation under unfair offers, implying that individuals experience greater perceived violations of fairness norms (Fehr and Camerer, 2007; Harlé *et al.*, 2012).

The emotion model posits that negative emotions provoked by unfair offers lead to rejections in the UG (Pillutla and Murnighan, 1996). fMRI studies have shown that the anterior insula and amygdala are more active for unfair offers than fair offers, suggesting that individuals might experience negative emotions such as aversion and anger when receiving unfair offers (Sanfey *et al.*, 2003; Gospic *et al.*, 2011; Harlé *et al.*, 2012).

The inequality aversion theory and strong reciprocity model explain individuals’ obedience to fairness norms from a motivational point of view, whereas the emotion model explores responses to fairness through an emotional lens. These three approaches have been used to explore the rationale behind individuals’ fairness preferences from different perspectives, reflecting the complexity of human social behavior.

## Social value orientation

Individual differences in fairness preferences represent an other-focused preference in social decision-making. Social value orientation (SVO) can explain a portion of this variance (Rilling and Sanfey, 2011). SVO is defined as the dispositional weights that individuals assign to outcomes for themselves and others in interdependent situations (McClintock, 1972; Murphy and Ackermann, 2014; Pletzer *et al.*, 2018). Two main types of SVO, prosocial and proself, are frequently distinguished in this population. Individuals with a prosocial orientation are willing to sacrifice their own interests to establish equal allocations and/or maximize joint outcomes, whereas individuals with a proself orientation focus on their personal interests in an effort to maximize their own outcomes while largely ignoring those of others (Liebrand, 1984; Van Lange and Kuhlman, 1994; Bogaert *et al.*, 2008). SVO has been regarded as a dispositional personality construct due to its temporal stability and situational consistency (Van Lange *et al.*, 1997; Balliet *et al.*, 2009; Murphy *et al.*, 2011), and it is expressed automatically (Kuss *et al.*, 2015). SVO is also closely related to the honesty–humility trait, which describes one’s tendency to be fair and honest and can effectively predict prosocial behavior in social dilemmas (Ashton and Lee, 2007; Hilbig *et al.*, 2014).

The integrative model of SVO suggests that prosocials pursue equality in outcomes and aim to maximize joint outcomes in interdependent situations, whereas proselfs seek to maximize personal outcomes and are less concerned with equality (Van Lange, 1999; De Cremer and Van Lange, 2001). Recent evidence has substantiated the claim that equality in outcomes is prosocials’ primary concern (Eek and Gärling, 2006; Van Lange *et al.*, 2013). Further, behavioral and neuroimaging studies have provided evidence for the integrative model. In the dictator game (a simplified version of the UG wherein the responder becomes a passive receiver of the proposer’s offer and therefore cannot reject it), prosocials allocate more resources to the receiver compared with proselfs, and this prosociality is expressed automatically (Cornelissen *et al.*, 2011). Moreover, prosocials are less likely to accept unfair offers than proselfs in the UG (Haruno *et al.*, 2014; Bieleke *et al.*, 2017). When asked to evaluate the desirability of reward pairs for the self and another in a reward-pair evaluation task, prosocials disliked large absolute differences in outcome allocations, whereas proselfs were unaffected by such differences and preferred larger personal rewards. Notably, the magnitude of differences in allocations was positively correlated with amygdala activation in prosocials but not in proselfs (Haruno and Frith, 2010). In a follow-up study, researchers found stronger amygdala activation for prosocials than proselfs in response to unfair offers in the UG (Haruno *et al.*, 2014). These findings suggest that prosocials possess stronger inequality aversion than proselfs in social decision-making and are more willing to punish unfair behavior at the expense of their own interests.

## Event-related potential components

Although studies involving fMRI have contributed greatly to identifying brain regions related to individuals’ responses to fairness (Haruno and Frith, 2010; Haruno *et al.*, 2014), little is known about the temporal characteristics of fairness processing among individuals with different SVOs in social interactions. The event-related potential (ERP) could reveal important insights about the precise timing of processes underlying cognitive functions given the measure’s excellent time resolution. Therefore, using the ERP in the present study, we aimed to examine the time characteristics of SVO in modulating fairness processing.

In ERP studies, several ERP components have often been used as biomarkers for outcome evaluation processing: the P2, the medial frontal negativity (MFN), and the P3. The P2 is a medial frontal positivity that peaks around ∼200–300 ms post-stimulus. It is associated with attentional capture (Potts *et al.*, 2004) and integration of motivational information, specifically reward information from the mesencephalic dopamine system, from an ongoing event (Potts *et al.*, 2006; Boudreau *et al.*, 2009); hence, the P2 is commonly considered indicative of automatic processing of a current event (Horat *et al.*, 2016). Prior study using DG has found that the P2 is greater for fair offers than for unfair offers (Wu *et al.*, 2011).

The MFN [also known as the feedback-related negativity (FRN)] is a fronto-central negativity that peaks at ∼200–350 ms post-outcome onset, presumably generated in the ACC, and represents a rapid processing of the current outcome in a ‘like–dislike’ dimension (Miltner *et al.*, 1997; Gehring and Willoughby, 2002; Yeung and Sanfey, 2004; Sambrook and Goslin, 2014; Pfabigan *et al.*, 2018). The MFN is typically more pronounced following negative outcomes than positive outcomes (Hajcak *et al.*, 2006; Paul and Pourtois, 2017; Sidarus *et al.*, 2017) and is sensitive to the degree to which an outcome deviates from expectations (Oliveira *et al.*, 2007; Bellebaum *et al.*, 2010; Alexander and Brown, 2011; Ferdinand *et al.*, 2012; Marciano *et al.*, 2018). During the UG, the MFN amplitude is more pronounced in response to unfair offers than to fair offers (Boksem and De Cremer, 2010; Hewig *et al.*, 2011; Massi and Luhmann, 2015; Peterburs *et al.*, 2017).

Following the MFN, the P3 is a centro-parietal positivity appearing in the 300–600 ms time window (Holroyd and Coles, 2002; Yeung and Sanfey, 2004; San Martín, 2012). The P3 encodes the valence and magnitude of an outcome, with a greater amplitude to an affectively positive than negative outcome and to a larger than smaller reward (Yeung and Sanfey, 2004; Sato *et al.*, 2005; Yeung *et al.*, 2005; Bellebaum and Daum, 2008; Glazer *et al.*, 2018). The P3 reflects allocation of attentional resources and the subjective importance of a desired outcome (Nieuwenhuis *et al.*, 2005; Polich, 2007; Mussel *et al.*, 2018). ERP studies in social decision-making have found that the P3 is also sensitive to the fairness of outcome allocation, exhibiting a larger amplitude for fair offers than for unfair offers (Wu *et al.*, 2011; Wang *et al.*, 2017a).

Recent ERP studies have investigated the SVO effect on outcome processing in various social contexts. Hu *et al.* (2017) found that individuals with distinct SVOs processed outcomes involving others differently; that is, prosocials were more concerned about others’ outcomes in interdependent situations than proselfs as revealed by the FRN, P3 and late positive component. The SVO effect was also observed in the Chicken Game. Compared with proselfs, prosocials considered non-reciprocated cooperation as the least desirable outcome as evidenced by an enhanced FRN (Wang *et al.*, 2017a). When compared with other social agents, prosocials were sensitive to others’ outcomes in self-gain and self-loss contexts, whereas proselfs were only interested in others’ outcomes in the self-gain context as indicated by distinct FRN patterns (Qi *et al.*, 2018). These findings suggest that SVO influences the processing of outcomes involving others, further supporting our supposition that SVO could modulate fairness processing in social decision-making through outcome-related ERP indicators.

In the present study, a multi-round, one-shot UG task was conducted with electroencephalography (EEG) recording. A typical UG involves two players: a proposer and a responder. First, the proposer suggests a division of a sum of money. The responder can then either accept the proposal, in which case the sum is allocated as suggested, or reject it, in which case neither player receives any money. The P2, MFN and P3 (representing different stages of outcome processing) were selected for analysis. Due to the characteristics of these ERP components, we hypothesized that the three components would be modulated by SVO for fair and unfair offers. In particular, we predicted that the fairness factor would be processed automatically in the prosocial group rather than the proself group as reflected by distinct P2 patterns. Further, we anticipated that the effect of fairness would be more prominent in the prosocial group than the proself group as indicated by the MFN and P3. These findings would provide direct evidence of individual variation in social values contributing to differences in time-dynamic characteristics underlying fairness processing.

## Methods

### Participants

Sixty young, healthy participants (32 women and 28 men) between 18 and 24 years of age (*M* = 19.55, s.d. = 1.92) were recruited from the university population. All participants were right-handed and had normal or corrected-to-normal vision. They had no history of neurological or psychiatric disorders, and none were taking psychoactive medication at the time of the experiment. Data from one female participant were excluded due to an insufficient number of trials after artifacts were removed (<20 trials, the minimal number of trials required for a stable ERP component; Marco-Pallares *et al.*, 2011). The final sample for analysis consisted of 59 participants (31 women and 28 men) with an average age of 19.51 years (s.d. = 1.91 years). An a priori sample size estimation was conducted using G*Power v.3.1 (Faul *et al.*, 2009). According to the analysis (*d *= 0.25, *α* = 0.05, *β* = 0.9, analysis of variance (ANOVA): repeated measures, within-between interaction), a total sample size of 46 participants was required to detect a reliable effect. Written informed consent was obtained from all participants. The experiment was performed in accordance with the Declaration of Helsinki and was approved by the Ethics Committee of the Department of Psychology, Renmin University of China.

### Procedure

#### Session 1: measurement of SVO.

In the first session, participants’ SVOs were measured using the Slider Measure (Murphy *et al.*, 2011), an efficient and simple measure of SVO with documented high reliability and excellent convergent validity (Anderl *et al.*, 2015) and significant predictive validity on social decision-making tasks (Bogaert *et al.*, 2008; Bieleke *et al.*, 2017). On this six-item measure, participants were asked to choose between several self-other payoff combinations. Based on their decisions, an SVO angle in a two-dimensional space consisting of one’s own payoff and others’ payoff was computed (Murphy *et al.*, 2011; Pletzer *et al.*, 2018). This measurement allowed for calculation of a continuous SVO score; then participants could, in principle, be grouped into corresponding categories based on their scores on prosocial or proself social preferences (Fiedler *et al.*, 2013; Pletzer *et al.*, 2018). The boundary between the two categories was 22.45°; angles below 22.45° indicated proselfs and greater angles indicated a more prosocial orientation (Murphy *et al.*, 2011; Murphy and Ackermann, 2014).

#### Session 2: the UG.

After measuring SVOs, participants received general instructions about the UG. Each participant played the UG repeatedly in a series of one-shot trials as a proposer and a responder. In the proposer role, each participant could divide 10 Chinese yuan into two shares (one for her-/himself and the other for the unknown responder) for 30 rounds. They were told that these offers would be stored in the database and used for future participants.

**Fig. 1.**
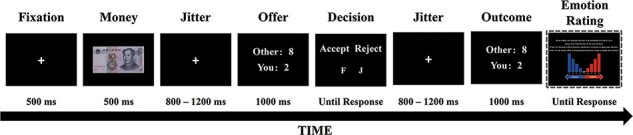
Illustration of a single trial of the multi-round one-shot UG task. Each trial began with presentation of a fixation cross for 500 ms followed by a picture of the 10-yuan bill for 500 ms. Then, a fixation cross was presented again for 800–1200 ms. Later, the offer screen appeared for 1000 ms, depicting a distributive outcome of 10 yuan between the respective proposer and the participant (responder). Participants decided to accept or reject the offer by pressing either the F or J key with their left or right index finger. After an interval of 800–1200 ms, outcome feedback was presented for 1000 ms. Participants were then asked to evaluate their emotional reaction to the offer on a 9-point scale. Emotion evaluation occurred randomly three times for each type of offer.

Afterward, participants played the UG in the responder role, while EEGs were recorded. In this crucial session, participants were informed they would receive 300 monetary offers proposed by volunteers and previous participants from the database, but they would receive only one offer from a particular proposer. In addition, participants were informed that the proposers would never know whether the offer was rejected or accepted. This explanation was fabricated to avoid learning and reputation effects, and the proposer session was conducted to render the task context more plausible. During the formal experiment, participants sat comfortably in a room shielded from sound and electrical fields, seated ∼80 cm from a 22-inch computer monitor. The entire task consisted of 300 trials in which all stimuli were displayed in the center of the computer screen. Figure 1 illustrates the time course of stimulus presentation in this UG task. Each trial began with a fixation cross appearing for 500 ms followed by a picture of a 10-yuan bill (2.6° × 1.3°) for 500 ms against a black background. A fixation cross was then presented again for 800 to 1200 ms. Later, the offer screen was presented for 1000 ms, depicting a distributive outcome of 10 yuan between the respective proposer and the participant (responder). Participants decided to accept or reject the offer at this point by pressing either the F or J key with their left or right index finger. After an interval of 800–1200 ms, feedback on the offer was presented for 1000 ms. Trials were separated by an inter-trial interval of 500 ms. In addition, participants were asked to evaluate their affective reaction to the currently received offer on a 9-point scale (1 = extremely negative and 9 = extremely positive) (Bradley *et al.*, 2001; Hewig *et al.*, 2011; Yang *et al.*, 2015). Emotion evaluation occurred three times randomly for each type of offer.

The entire task was divided into four blocks of 75 trials each with a brief break between blocks. Each block consisted of 30 fair trials (15 offers of 5–5 and 15 offers of 4–6), 30 unfair trials (15 offers of 1–9 and 15 offers of 2–8) and 15 moderately unfair offers (3–7). Unbeknownst to the participants, all offers were generated by the computer program rather than actual people. Therefore, when a participant acted as the responder, each type of offer was presented in a random sequence. Before the formal task, participants completed 10 practice trials to familiarize themselves with the UG task. The entire task lasted for ∼30 min. Before the EEG task, all participants were informed they would be paid 30 Chinese yuan for their participation plus the cumulative outcome based on their decisions during the task. Upon finishing the experiment, each participant was paid roughly 70 Chinese yuan regardless of their decisions in the UG task. Participants were also asked about the plausibility of the cover story, and no participants expressed suspicion about it. Stimulus presentation and behavioral data acquisition were conducted using E-Prime 2.0 software (PST, Inc., Pittsburgh, PA, USA).

### EEG recording and preprocessing

EEG data were recorded using 64 cap-mounted tin electrodes arranged according to the 10–20 international placement system (Neuroscan Inc., Herndon, VA, USA), with an online reference to the left mastoid and an offline re-reference to the averaged mastoid electrodes. A horizontal electro-oculogram (EOG) was recorded from electrodes placed 1.5 cm laterally to the outer canthi of both eyes. A vertical EOG was recorded from electrodes placed above and below the left eye. All inter-electrode impedance was kept below 5 kΩ during recording. Signals were amplified using a 0.01–100 Hz band-pass filter and continuously sampled at 500 Hz/channel. Offline analysis of EEG data was performed using Neuroscan software (Scan 4.5, NeuroScan, Inc., Herndon, VA, USA). Ocular artifacts were removed from EEGs using a regression procedure implemented in Scan 4.5 software (Semlitsch *et al.*, 1986). All EEG data were digitally low-pass filtered at 30 Hz (24 dB/oct) and segmented into epochs time-locked to the onset of offer presentation, beginning 200 ms before offer onset and continuing for 1000 ms. Data were then baseline-corrected according to the 200 ms pre-offer period. Epochs containing artifacts exceeding ± 70 μV were excluded from subsequent analyses. Then, epochs were averaged separately for each condition of each participant.

### Statistical analyses

#### Behavioral data.

Participants’ SVO scores were analyzed using an independent-samples *t*-test. Acceptance rates (ARs) and emotion ratings were subjected to mixed two-way repeated-measures ANOVA with SVO type (proself *vs* prosocial) as a between-subjects factor and fairness (fair *vs* unfair) as a within-subject factor. Trials with a moderate unfair offer (3–7) in this task functioned as fillers and thus excluded from analyses; previous studies reported that responders held diverse opinions about whether this offer could be considered fair (Halko *et al.*, 2009; Hewig *et al.*, 2011; Luo *et al.*, 2014), resulting in difficulty classifying this type of offer.

#### ERP data.

**Fig. 2.**
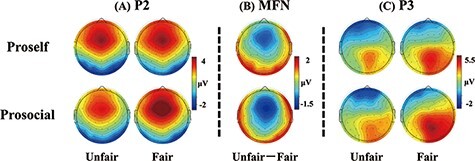
Topographical voltage distributions of the P2 (200–270 ms) and P3 (400–600 ms) for fair and unfair conditions and topographies of voltage differences between unfair and fair offers in the MFN time interval (290–370 ms) for SVO groups, respectively.

Analyzed ERP components included the P2, MFN and P3. For statistical analyses, based on previous studies and inspection of the grand-averaged waveforms, amplitudes of the P2, MFN and P3 were quantified as mean values within the time windows of 200–270 ms (Potts *et al.*, 2006; Luo *et al.*, 2015), 290–370 ms (Polezzi *et al.*, 2008; Wu *et al.*, 2011; Massi and Luhmann, 2015) and 400–600 ms (Luo *et al.*, 2014; Peterburs *et al.*, 2017; Wang *et al.*, 2017a) following the onset of the offer stimulus, respectively. Compared to peak measures, mean measures are less sensitive to high-frequency noise and not biased by the noise level, and thus, could make the statistical results more reliable (Luck, 2014). Based on prior studies (Gehring and Willoughby, 2002; Yeung and Sanfey, 2004; Luo *et al.*, 2015) and scalp topographies of each ERP component (Figure 2), the P2 and MFN were calculated at the FCz site, and the P3 was calculated at the Pz electrode site where they reached maximum amplitudes. The ERP amplitudes were subjected to mixed two-way repeated-measures ANOVA with SVO type (proself *vs* prosocial) as a between-subjects factor and fairness (fair *vs* unfair) as a within-subject factor.

#### Neural-behavioral correlation.

To evaluate the relationships between behavioral responses and neural activities, Pearson correlation coefficients were calculated between fairness enforcement performance and brain activities.

#### Mediation analysis.

Mediation analysis was conducted using the SPSS version of the INDIRECT macro (Preacher and Hayes, 2008), taking the SVO score as the independent variable, d-MFN (MFN for unfair offers minus MFN for fair offers) as the mediator and ARs for unfair offers as the outcome variable.

The significance level was set at 0.05 for all analyses. The Greenhouse–Geisser correction was conducted to account for sphericity violations whenever appropriate. Post-hoc testing of significant main effects was applied with Bonferroni adjustments. Partial eta-squared (*η*_p_^2^) values were calculated to indicate the effect size in ANOVA models, with 0.05 representing a small effect, 0.1 representing a medium effect and 0.2 representing a large effect (Cohen, 1973). Behavioral and ERP data were analyzed statistically using SPSS software (version 25.0; IBM Corp., Armonk, NY, USA).

## Results

### Behavioral results

#### SVO scores and test-retest reliability.

The SVO scores of 59 participants in this study ranged from −7.80° to 37.85°, of which 30 were proselfs (score < 22.45°) and 29 were prosocials (score > 22.45°). Mean (±s.d.) SVO scores of prosocials were significantly higher than those of proselfs (31.09 ± 7.14° *vs* 13.29 ± 4.51°; *t*(1, 57) = 11.41, *p* < 0.001, Cohen’s *d* = 3.02). To test the reliability of SVO, 1 year after the first measurement, all participates were contacted by e-mail and asked to complete the SVO Slider Measure again. Fifty-one participants (25 proselfs and 26 prosocials) completed the SVO Slider Measure again. Forty-six of them were divided into the same SVO category into two times of the Slider Measure, yielding a consistency of 90%. Further the correlation between the resulting angles from the test–retest SVO Slider Measure was *r* = 0.885. The results of test–retest reliability in this study are consistent with the finding of the previous study (Murphy *et al.*, 2011).

#### ARs.

**Fig. 3.**
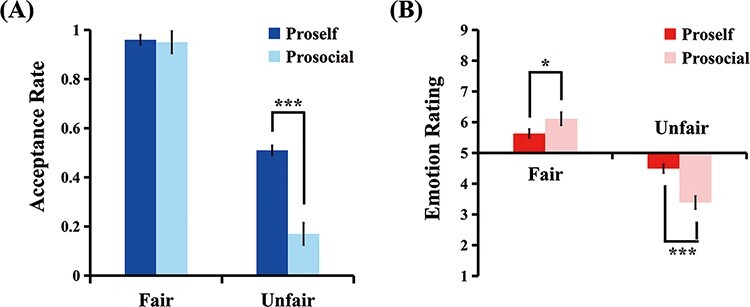
Histogram of interaction effect between SVO type and fairness on ARs (A) and emotion ratings (range from 1–9) (B). Error bars indicate standard error of the mean (SEM). *P < 0.05, ***P < 0.001.

Figure 3A illustrates the ARs of offers. A significant main effect of fairness was confirmed, *F*(1,57) = 213.32, *P *< 0.001, *η*_p_^2^ = 0.79, with lower ARs for unfair offers than fair offers (0.34 ± 0.37 *vs* 0.96 ± 0.11). The main effect of SVO type was also significant, *F*(1, 57) = 12.98, *P *< 0.001, *η*_p_^2^ = 0.19, with lower ARs in the prosocial group than the proself group (0.56 ± 0.44 *vs* 0.74 ± 0.36). Notably, the interaction effect of SVO type × fairness was significant, *F*(1, 57) = 15.91, *P *< 0.001, *η*_p_^2^ = 0.22. Simple-effects analysis of the interaction effect demonstrated that prosocials were more likely than proselfs to reject unfair offers (0.17 ± 0.26 *vs* 0.51 ± 0.38), *F*(1, 57) = 15.98, *P *< 0.001, *η*_p_^2^ = 0.22. Conversely, no significant difference was found for fair offers (0.95 ± 0.11 *vs* 0.96 ± 0.12), *F*(1, 57) = 0.07, *P* = 0.79.

#### Emotion ratings.

Figure 3B illustrates participants’ emotion ratings of offers. For emotional ratings, the main effect of fairness was significant, *F*(1, 57) = 253.90, *P *< 0.001, *η*_p_^2^ = 0.82, with more negative emotional reactions to unfair offers than to fair offers (3.91 ± 1.04 *vs* 5.88 ± 0.86). The interaction effect of SVO type × fairness was also significant, *F*(1, 57) = 38.32, *P *< 0.001, *η*_p_^2^ = 0.40. Simple-effects analysis of the interaction effect revealed that prosocials were more prone than proselfs to experiencing more positive reactions to fair offers (6.11 ± 0.91 *vs* 5.66 ± 0.77; *F*(1, 57) = 4.30, *P* = 0.043, *η*_p_^2^ = 0.07) and to experiencing more negative reactions than proselfs to unfair offers (3.37 ± 0.69 *vs* 4.45 ± 1.06), *F*(1, 57) = 21.48, *P* < 0.001, *η*_p_^2^ = 0.27.

### ERP results

#### P2.

**Fig. 4.**
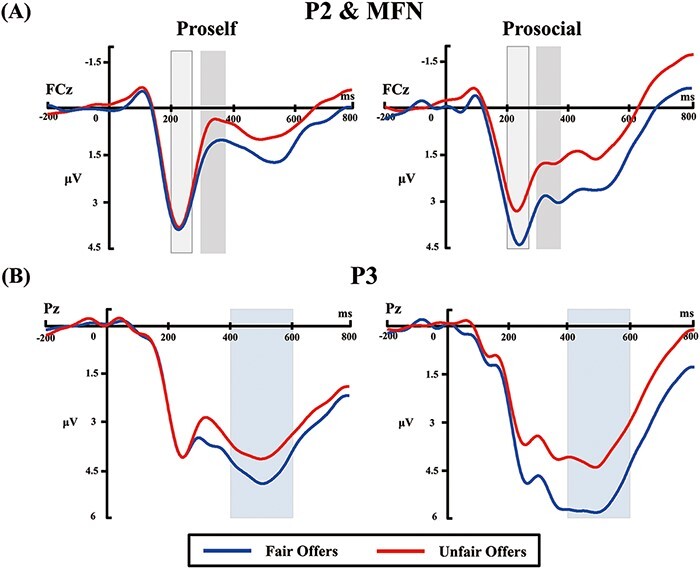
Grand-average ERP waveforms from FCz and Pz electrode sites for the SVO groups, respectively. (A) Light gray and darker gray areas denote time windows of 200–270 ms and 290–370 ms in which peak amplitudes of the P2 and MFN were measured, respectively. (B) Light blue areas denote the time window (400–600 ms) in which the mean amplitude of the P3 was measured.

Figure 4A depicts grand-average ERP waveforms at the FCz electrode site. The P2 and MFN were measured at the FCz site. For the P2 amplitude, the main effect of fairness was significant, *F*(1, 57) = 17.41, *P *< 0.001, *η*_p_^2^ = 0.23, indicating that fair offers elicited a larger P2 compared with unfair offers (3.85 μV *vs* 3.30 μV). Importantly, a significant interaction effect emerged between SVO type and fairness, *F*(1, 57) = 9.13, *P *= 0.004, *η*_p_^2^ = 0.14. A simple-effects analysis was conducted to investigate the two-way interaction; results showed that the fairness effect was only significant for prosocials, indicating a larger P2 in response to fair offers than unfair offers (4.08 μV *vs* 3.14 μV), *F*(1, 57) = 25.45, *P *< 0.001, *η*_p_^2^ = 0.31. In contrast, the fairness effect on the P2 was not significant for proselfs (3.62 μV *vs* 3.47 μV), *F*(1, 57) = 0.67, *P *= 0.415. The SVO difference of this fairness effect is displayed in a topographic map of the P2 (Figure 2A).

#### MFN.

As to the MFN amplitude, the main effect of fairness was significant, *F*(1, 57) = 58.91, *P *< 0.001, *η*_p_^2^ = 0.51, indicating that unfair offers elicited a more negative-going MFN compared with fair offers (1.19 μV *vs* 2.23 μV). Moreover, a significant interaction effect was observed between SVO type and fairness, *F*(1, 57) = 8.47, *P *= 0.005, *η*_p_^2^ = 0.13. Consequently, a simple-effects analysis was conducted to investigate the significant interaction. Findings indicated that although the MFN was more negative-going in response to unfair offers than fair offers in both SVO groups, the fairness effect on the MFN was more prominent in the prosocial group (1.70 μV *vs* 3.14 μV), *F*(1, 57) = 55.10, *P *< 0.001, *η*_p_^2^ = 0.49 than the proself group (0.68 μV *vs* 1.33 μV), *F*(1, 57) = 11.07, *P *= 0.002, *η*_p_^2^ = 0.16. The SVO difference in this fairness effect is shown in a topographic map of the MFN (Figure 2B).

#### P3.

Figure 4B presents grand-average ERP waveforms at the Pz electrode site. The main effect of fairness on the P3 amplitude was significant, *F*(1, 57) = 42.55, *P *< 0.001, *η*_p_^2^ = 0.43, indicating that fair offers elicited a larger P3 than unfair offers (4.63 μV *vs* 3.64 μV). A significant interaction effect emerged between SVO type and fairness, *F*(1, 57) = 6.38, *P *= 0.014, *η*_p_^2^ = 0.10. Thus, a simple-effects analysis was conducted to explore the significant interaction effect. Results revealed that the P3 was larger in response to fair offers than unfair offers in both SVO groups, whereas the fairness effect on the P3 (fair minus unfair) appeared more prominent in the prosocial group (5.04 μV *vs* 3.67 μV), *F*(1, 57) = 40.27, *P *< 0.001, *η*_p_^2^ = 0.41 than the proself group (4.21 μV *vs* 3.61 μV), *F*(1, 57) = 8.12, *P *= 0.006, *η*_p_^2^ = 0.13. This analysis further revealed that the P3 amplitudes elicited by fair offers were significantly larger in the prosocial group than the proself group (5.04 μV *vs* 4.21 μV), *F*(1, 57) = 4.28, *P *= 0.43, *η*_p_^2^ = 0.07, whereas the P3 amplitudes elicited by unfair offers were comparable between SVO groups (3.67 μV *vs.* 3.61 μV), *F*(1, 57) = 0.02, *P *= 0.894. The SVO difference of the fairness effect is displayed in a topographic map of the P3 (Figure 2C).

### Results of neural-behavioral correlation

Next, we examined whether increase in individuals’ SVO scores was associated with a corresponding decrease in ARs for unfair offers. A Pearson correlation analysis was conducted between SVO scores and ARs for unfair offers. The results revealed that SVO scores were negatively correlated with ARs for unfair offers, *r* = −0.50, *P* < 0.01. To address the potential contribution of neural activities to decreased ARs for unfair offers, we examined whether the P2, MFN or P3 effects correlated with ARs for unfair offers. Results showed only the MFN effect (d-MFN) was positively correlated with ARs for unfair offers, *r* = 0.59, *p* < 0.01; however, no other correlations survived, *ps* > 0.102.

### Results of mediation analysis

**Fig. 5.**
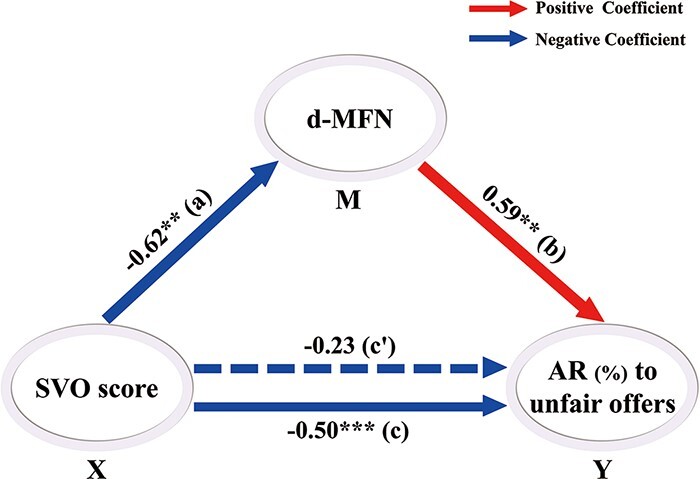
Mediation effect: the effect of SVO scores on ARs for unfair offers was significantly reduced when the d-MFN was included in the regression model. Estimates are standardized values. ***P < 0.001.

In this study, given an SVO effect on both the d-MFN and ARs for unfair offers, we conducted a mediation analysis to explore whether the SVO effect on participants’ responses to unfairness was mediated by the d-MFN. The findings confirmed that the effect of SVO on ARs for unfair offers was mediated by the d-MFN, Sobel test: *t* = −2.92, *P* = 0.003. A stepwise regression excluding the factor of SVO was no longer significant when d-MFN was introduced, *β* = −0.23, *p* = 0.099, as compared with initial coefficient, *β* = −0.50, *P* < 0.001, suggesting that the effect of SVO on d-MFN acted as a full mediator of the effect of SVO on ARs for unfair offers (Figure 5). Bootstrapping results indicated that this mediation effect was different from zero with 95% confidence (number of bootstrap resamples = 5000; *β* = −0.83, confidence intervals = −1.55 to −0.21). Confidence intervals for indirect effect were bias-corrected (Preacher and Hayes, 2008).

## Discussion

The present study investigated the modulating effect of SVO on individuals’ fairness processing in the UG task and explored the temporal characteristics of neural activities underlying this issue through the ERP technique. As expected, the results demonstrated that individuals with distinct SVOs responded differently to fairness in the UG and revealed the neural underpinnings of SVO modulation of fairness processing. Prosocials appeared more satisfied with fair offers and more upset about being treated unfairly than proselfs, in which case prosocials were less likely than proselfs to accept unfair offers. Electrophysiologically, three ERP indicators (the P2, MFN and P3), revealed group differences in fairness processing. Compared with proselfs, prosocials exhibited automatic processing to the fairness factor as evidenced by a significant difference in the P2 for fair and unfair offers. Furthermore, prosocials exhibited deeper processing to fairness in later time windows, reflected in the MFN and P3. Moreover, the fairness effect on the MFN (d-MFN) mediated the relationship between individuals’ SVOs and behavioral responses to unfairness in the UG. We discuss the implications of these findings in the subsequent sections.

### SVO modulates individuals’ behavioral responses to fairness in social decision-making

In the present study, unfair offers induced negative affective responses from responders, concomitant with findings from prior research (Damasio *et al.*, 2000; van’t, Kahn, Sanfey, and Aleman, 2006; Xiao and Houser, 2005; Hewig *et al.*, 2011; Gilam *et al.*, 2015). Also, the behavioral results replicated a well-established behavioral pattern wherein people are likely to reject unfair offers in the UG (Güth *et al.*, 1982; Nowak *et al.*, 2000; Holper *et al.*, 2018).

The abovementioned affective and behavioral responses were modulated by SVO. Studies have found that compared with proselfs, prosocials tend to prefer equal outcomes in economic decision-making, suggesting that fairness is more important for prosocials than for proselfs (Van Lange, 1999; Eek and Gärling, 2006); therefore, fair offers induced more positive emotional experiences for prosocials. However, unfair offers violated their inner norms of pursuing equality in outcomes and thus induced more negative reactions for prosocials than for proselfs, as indicated in fMRI studies wherein amygdala activation was significantly stronger for prosocials than for proselfs when encountering unequal outcomes (Haruno and Frith, 2010; Haruno *et al.*, 2014). The trend of prosocials rejecting unfair offers more frequently than proselfs was also consistent with previous research, suggesting that prosocials are more willing to sacrifice personal benefits to retaliate against unfair behaviors and enforce fairness norms (Haruno *et al.*, 2014; Bieleke *et al.*, 2017). Unsurprisingly, as the correlation result implied, individuals’ SVO could predict the likelihood of rejecting unfair offers in the UG, with a more prosocial SVO indicating a higher chance of rejection.

### SVO modulates the early stage of fairness processing

The first stage of fairness processing is represented by the P2, which reflects an initial, automatic and low-level processing of outcomes (McPartland *et al.*, 2011; Van der Molen *et al.*, 2014; Luo *et al.*, 2015). Research has found that the P2 is sensitive to motivational information of stimuli, with larger amplitudes associated with outcomes that meet motivational goals (Potts *et al.*, 2006; Boudreau *et al.*, 2009). In this study, the fairness effect on the P2 was modulated by SVO: the fairness effect was only significant for prosocials, who demonstrated a larger P2 in response to fair offers than to unfair offers. Considerable evidence has suggested that SVO is expressed automatically, and prosocials and proselfs seem to possess different intrinsic motivations (Cornelissen *et al.*, 2011; Kuss *et al.*, 2015). Specifically, prosocials tend to internalize fairness norms as an intrinsic principle (Cornelissen *et al.*, 2011). An equal outcome is therefore optimal in prosocials’ decision-making pursuits, and they process fairness in outcome allocations automatically as an intuitive response (Van Lange *et al.*, 2013). Proselfs prioritize maximizing self-interest as their decision-making objective, which leads them to process their own benefits preferentially (Balliet *et al.*, 2009).

Compared with proselfs, prosocials were more sensitive to fairness in outcomes given a stronger fairness principle; prosocials also demonstrated automatic processing of fairness at the early stage. Fair offers in the UG met prosocials’ goal of pursuing fairness and held stronger motivational salience, thus eliciting a larger P2 than unfair offers. Comparatively, due to their primary processing of self-benefits, proselfs were not sensitive to outcome fairness in the early processing stage; therefore, the P2 patterns for fair and unfair offers did not reveal a difference in the proself group. These results support behavioral and fMRI findings from previous studies indicating that SVO plays an important role in automatic intuitive processing in fairness-related decision-making (Haruno *et al*., 2010; Cornelissen *et al.*, 2011; Haruno *et al.*, 2014; Bieleke *et al.*, 2017).

### SVO modulates the middle stage of fairness processing

The middle stage of fairness processing is represented by the MFN, a critical ERP component related to outcome processing; it reflects a rapid, coarse and semi-automatic coding of ongoing events (Yeung and Sanfey, 2004; Hajcak *et al.*, 2006; Leng and Zhou, 2010; Hu *et al.*, 2017; Kimura *et al.*, 2018;). Consistent with prior research, unfair offers elicited a more negative-going MFN than fair offers in this study, implying that individuals considered unfair offers an unfavorable outcome that violated social norms (Polezzi *et al.*, 2008; Boksem and De Cremer, 2010; Hewig *et al.*, 2011; Van Der Veen and Sahibdin, 2011; Luo *et al.*, 2014; Mothes *et al.*, 2016). We found the fairness effect on the MFN to be modulated by SVO; specifically, the fairness effect was more prominent in the prosocial group than in the proself group, indicating that prosocials processed fairness in outcomes more deeply than proselfs. Interestingly, the fairness effect on the MFN (d-MFN) mediated the relationship between SVO and behavioral responses to unfair offers in the UG. That is, a more prosocial SVO led individuals to exhibit a stronger fairness effect on the MFN, which made them less likely to accept unfair offers.

Studies using fMRI have shown that receiving unfair offers is associated with increased activation in the ACC, a brain region involved in the processing of outcomes that deviate negatively from expectations (Sanfey *et al.*, 2003; Amiez *et al.*, 2005; Matsumoto *et al.*, 2007; Boksem and De Cremer, 2010; Harlé *et al.*, 2012). According to the reinforcement learning (RL) theory of the MFN, the key function of the ACC is the adaptive control of behavior, and the ACC learns how to perform its function better from discrepancies between actual and expected positive outcomes (Holroyd and Coles, 2002; Holroyd *et al.*, 2008). Therefore, the RL theory postulates that when events are worse than expected, the decrease of dopamine activity trains the ACC to adjust the control of the motor system with an enhanced MFN, which indicates an unexpected and undesirable outcome (Holroyd and Coles, 2002; San Martín, 2012). In the UG task, prosocials regarded fair offers as favorable ones owing to the higher earnings and fairness, which elicited a gentler MFN. However, unfair offers were regarded as unfavorable outcomes because of their lower benefit and equality, which were much worse than their expected ones and thus elicited a larger MFN. In contrast, proselfs mainly focused on their personal interests in the UG and put less value on the fairness embedded in the offers. Therefore, the fairness effect of the MFN in the proself group was weaker than that in the prosocial group.

On the basis of the RL theory, the predicted response–outcome (PRO) model proposed by Alexander and Brown (2010, 2011) suggests that the general role of the ACC is to predict the likely outcomes of actions and to signal unexpected non-occurrences of events. Therefore, the MFN is larger when an actual outcome does not match the expected one (Ferdinand *et al.*, 2012). According to the PRO model, prosocials in the present study had strong internalized fairness norms and held higher expectation for fair offers than proselfs. Therefore, fair offers were expected and elicited a gentler MFN, whereas unfair offers deviated far from their expectations and elicited an enhanced MFN. Consequently, the prosocial group demonstrated a stronger fairness effect on the MFN than proself group did.

On the other hand, people tend to form expectations of partners’ behavior in social interactions, especially in situations in which information about other social agents is lacking (Kelley and Stahelski, 1970). The Structural Assumed Similarity Bias model proposes that individuals with different SVOs are prone to projecting their own dispositions onto others and thus expect others to be similar to themselves (Kuhlman and Wimberley, 1976; Ross *et al.*, 1977; Declerck and Bogaert, 2008; Pletzer *et al.*, 2018). Accordingly, prosocials expected more fair behavior from their partners in the UG, and this false consensus effect elicited a stronger fairness effect on the MFN. Conversely, proselfs assumed their partners were similar to themselves and thus had lower expectations for fair behavior, generating a weaker fairness effect on the MFN relative to prosocials. Subsequently, according to the goal-expectation hypothesis, expectations about others’ behavior can influence individuals’ decision-making (Pruitt and Kimmel, 1977; Komorita *et al.*, 1992). Therefore, in this study, a more prosocial SVO caused individuals to possess higher expectations for fair outcomes. When confronted with outcomes violating this expectancy, participants were more likely to reject unfair offers. This mediating effect has been partially supported by previous studies in which the anticipated cooperation mediated the relationship between SVO and cooperation in social dilemmas (Balliet *et al.*, 2009; Renkewitz *et al.*, 2011; Balliet and Van Lange, 2013; Pletzer *et al.*, 2018).

In addition to evaluating whether outcomes deviated from expectations, the ACC is recruited by affectively aversive processing of social pain (Eisenberger, 2012; Rotge *et al.*, 2015; Dalgleish *et al.*, 2017; Schmälzle *et al.*, 2017; Chester *et al.*, 2018). The motivational/affective theory of the MFN holds that the MFN reflects a motivational/affective evaluation of an outcome event, and this evaluation is particularly sensitive to a negative outcome (Gehring and Willoughby, 2002; Pailing and Segalowitz, 2004). That is, the MFN may represent evaluation of the subjective value of an outcome (Luo *et al.*, 2014). Thus, for prosocials who value fairness, unfair offers that violated fairness norms elicited stronger negative feelings, reflected in more pronounced MFN responses compared with proselfs (who had less regard for such fairness norms). This result was in line with a previous ERP study in which the fairness effect on the MFN was stronger in participants with high moral identity (Boksem and De Cremer, 2010). Moreover, on the basis of the emotion model of fairness processing, negative emotions induced by perceived unfairness have been found to be predictive of rejection behaviors in the UG (Pillutla and Murnighan, 1996; Sanfey *et al.*, 2003). Accordingly, individuals with higher SVO scores (i.e. who are more prosocial) may experience stronger negative reactions to unfair offers and be more prone to reject such offers to punish unfair behaviors.

### SVO modulates the late stage of fairness processing

The late stage of fairness processing is represented by the P3, another important ERP component reflecting elaborative and sustained processing of a current outcome (Yeung and Sanfey, 2004; Philiastides *et al.*, 2010; Hu *et al.*, 2017). Research has indicated that the P3 is related to processes of attentional allocation and high-level motivational or affective evaluation, namely in signaling the subjective awareness and importance of a desired outcome (Schupp *et al.*, 2004; Nieuwenhuis *et al.*, 2005; Wu and Zhou, 2009; Mussel *et al.*, 2018). Similar to earlier research, fair offers evoked a larger P3 than unfair offers in this study; thus, compared with unfair offers, fair offers were considered desirable outcomes consistent with individuals’ fairness preference and allocated more attentional resources from them (Wu *et al.*, 2011; Qu *et al.*, 2013; Wang *et al.*, 2017a). Remarkably, the fairness effect on the P3 was modulated by SVO: fair offers evoked stronger P3 responses among prosocials than proselfs.

As proposed in the integrative model of SVO, relative to proselfs, prosocials generally assign greater weight to equality in outcomes and outcomes for others (Van Lange, 1999; Van Lange *et al.*, 2013). Scholars have also found that prosocials prefer equal outcomes compared with proselfs when making decisions, and this fairness preference is so strong that prosocials even prefer to make personal sacrifices to attain equal outcomes (Eek and Gärling, 2006). As such, in the current study, fair offers in the UG carried different meanings for prosocials and proselfs. For proselfs, a fair offer represented a more profitable outcome that met these individuals’ motivation to maximize their self-interests, thus evoking more attentional resources as reflected by enhanced P3 responses. For prosocials, a fair offer indicated greater benefit along with adherence to fairness norms, fulfilling their primary goal of pursuing equality in outcomes. Prior fMRI studies revealed that individuals reacted to fairness with a positive hedonic response, and fairness preferences were associated with reward-based regions of the brain including the ventral striatum, vmPFC and orbitofrontal cortex (Tabibnia and Lieberman, 2007; Tabibnia *et al.*, 2008; Tricomi *et al.*, 2010). Therefore, compared with proselfs, a fair offer appeared more rewarding and desirable for prosocials, who placed greater subjective importance on such outcomes. Accordingly, the P3 response for fair offers was stronger in the prosocial group, lending support to inequality aversion theory (Chen and Vazsonyi, 2011; Harlé *et al.*, 2012).

In summary, this study unveiled a modulating effect of SVO on individuals’ fairness processing during social decision-making using an ERP technique. Behaviorally, prosocials were more willing to reject unfair offers and had stronger negative emotional experiences than proselfs; prosocials were more satisfied with fair offers. Electrophysiologically, relative to proselfs, prosocials exhibited automatic intuitive processing of fairness at an early stage represented by the P2, suggesting that SVO reflects stable individual differences in an inherent sense of outcome fairness. In the middle processing stage, unfairness greatly violated prosocials’ expectations and triggered stronger negative emotional reactions than in proselfs as depicted by the MFN. Importantly, participants’ SVO affected their rejection of unfair offers via the d-MFN evoked by unfair and fair offers, which might reveal neural mechanisms underlying the SVO effect on individuals’ behavioral reactions to unfairness. Correspondingly, compared with proselfs, fair outcomes met prosocials’ decision-making motivation and attracted more of their attention as indicated by the P3. These findings shed light on differences in the behavioral patterns and temporal characteristics of neural activities among individuals with distinct SVOs in fairness processing. Such results help extend the literature on behavioral responses and neuroimaging, offering compelling evidence for classic models related to fairness norms relevant to numerous perspectives and processing stages.

### Limitations and outlook for subsequent research

There are several limitations in our study. First, we only considered disadvantageous inequality (DI) as unfairness in the present study. However, advantageous inequality (AI) also plays an important role in fairness-related decision-making. Although human beings show aversion to both DI and AI, previous studies have found that individuals strongly oppose DI, while their responses to AI are relatively modest (Falk and Fischbacher, 2006; Yu *et al.*, 2014). In addition, studies have shown that evaluating AI requires more cognitive resources, such as reputation management and sustainability of cooperation, than evaluating DI (van den Bos *et al.*, 2006; Fliessbach *et al.*, 2012; Gao *et al.*, 2018). Since individuals’ SVO has an impact on the trade-off between economic interests and fairness norm enforcement, it is meaningful to explore the SVO effect of individual processing AI offers in the UG.

Second, we only focused on highly unfair offers and fair offers and excluded the moderately unfair offer (3–7 offer) in this study. The moderately unfair condition is also worth exploring due to its fuzziness and uncertainty (Polezzi *et al.*, 2008; Duek *et al.*, 2014; Wang *et al.*, 2017a). Are prosocials and proselfs’ evaluation criteria for moderately unfair schemes different? Could this diversity affect individuals’ response patterns? These issues are worthy of further exploration in the future.

Finally, other personal characteristics, such as gender differences, should also be taken into account in future work. Previous studies have shown that gender has an impact on the level of trust in individuals such that women trust others more than men in economic transactions (Chaudhuri *et al.*, 2013; Haselhuhn *et al.*, 2015). In addition, signals of trustworthiness influence the relationship between SVO and cooperation: compared to proselfs, prosocials are more sensitive to information signaling trustworthiness of others (Bogaert *et al.*, 2008). Therefore, it would be interesting to investigate how gender and SVO interactively affect individuals’ fairness processing in social decision making.
